# EndoV does not measurably affect TadA-dependent A-to-I RNA editing in *Escherichia coli* under exponential-growth conditions in rich medium

**DOI:** 10.1128/spectrum.01302-26

**Published:** 2026-07-06

**Authors:** Ofir Fargeon, Dan Bar-Yaacov

**Affiliations:** 1 The Shraga Segal Department of Microbiology, Immunology, and Genetics, Ben-Gurion University of the Negev 26732, Be'er Sheva, Israel; Forschungszentrum Jülich GmbH, Juelich, Germany; Northwest A&F University, Yangling, China

**Keywords:** A-to-I RNA editing, bacteria, TadA, EndoV, *nfi*, *Escherichia coli*

## Abstract

Adenosine-to-inosine (A-to-I) mRNA editing changes the genetic information post-transcriptionally and was only recently reported to occur in bacteria. Here, we examined whether endonuclease V (EndoV; encoded by *nfi*) cleaves inosine-containing RNAs *in vivo* and thereby influences the abundance and fate of A-to-I-edited mRNAs in *Escherichia coli*. We generated an *nfi* loss-of-function mutant carrying a premature stop codon and performed RNA sequencing alongside the isogenic wild-type strain. We observed that global and site-specific editing occurrence or levels in both mRNAs and tRNAs were indistinguishable between strains. Moreover, overexpression of EndoV did not affect the number of edited sites, motif enrichment, or editing levels compared with a control strain overexpressing mCherry. Our findings suggest that, in contrast to human EndoV, bacterial EndoV does not regulate the steady-state pool of edited mRNAs in *E. coli* under nutrient-rich, exponential-growth conditions *in vivo*.

**IMPORTANCE:**

Adenosine-to-inosine (A-to-I) mRNA editing is an emerging regulatory layer in bacteria, but the factors that act on edited transcripts are largely unknown. Endonuclease V (EndoV) was a prime candidate because it cleaves inosine-containing nucleic acids and can act on inosine-containing RNA *in vitro*. By combining loss-of-function and overexpression of EndoV with genome-wide RNA editing measurements, we show that EndoV does not measurably influence TadA-dependent A-to-I mRNA editing in *Escherichia coli* under standard laboratory conditions. This negative result is important because it rules out a natural effector candidate and redirects attention to other bacterial pathways that may process edited RNAs. Our work, therefore, sharpens mechanistic models for bacterial RNA editing and helps focus future searches for its regulators and physiological roles.

## OBSERVATION

Adenosine-to-inosine (A-to-I) RNA editing is a post-transcriptional RNA modification that generates RNA sequences differing from the DNA templates from which they are transcribed (1–3). A-to-I RNA editing is essential for various cellular processes across diverged eukaryotic organisms (3–10). Until recently, bacterial RNA editing had been reported only in arginine tRNA (tRNA-Arg2; anticodon ACG), where it is catalyzed by the tRNA-specific adenosine deaminase TadA (11).

We previously reported that, in *Escherichia coli*, TadA edits bacterial mRNA in addition to its canonical tRNA substrate (12). Since then, A-to-I mRNA editing has been identified in additional bacterial species, including human pathogens (13–17). Notably, TadA was shown to recognize a defined core sequence motif (UACG) together with an RNA secondary structure resembling the anticodon stem-loop of tRNA-Arg2 (11, 12). Moreover, A-to-I editing has been shown to recode protein sequences in bacteria and to be modulated by factors such as RNA stability and growth phase (13–16). However, the factors governing its occurrence remain poorly understood.

Endonuclease V (EndoV) is a ubiquitous enzyme that cleaves the second phosphodiester bond 3′ to inosine (18). In *E. coli*, EndoV is encoded by the *nfi* gene and primarily initiates DNA repair at deaminated adenines (19). In humans, however, EndoV has evolved to cleave inosine-containing RNAs (20, 21). Recently, *E. coli* EndoV was shown to cleave inosine-containing RNA *in vitro*, providing a powerful tool for inosine detection in RNA (22). Thus, here, we examined whether EndoV can cleave inosine-containing RNA *in vivo* and thereby influence the landscape and fate of A-to-I-edited transcripts in bacteria.

## RESULTS

### Introducing a premature stop codon at EndoV does not affect *in vivo* A-to-I RNA editing occurrence or levels

**Fig 1:**
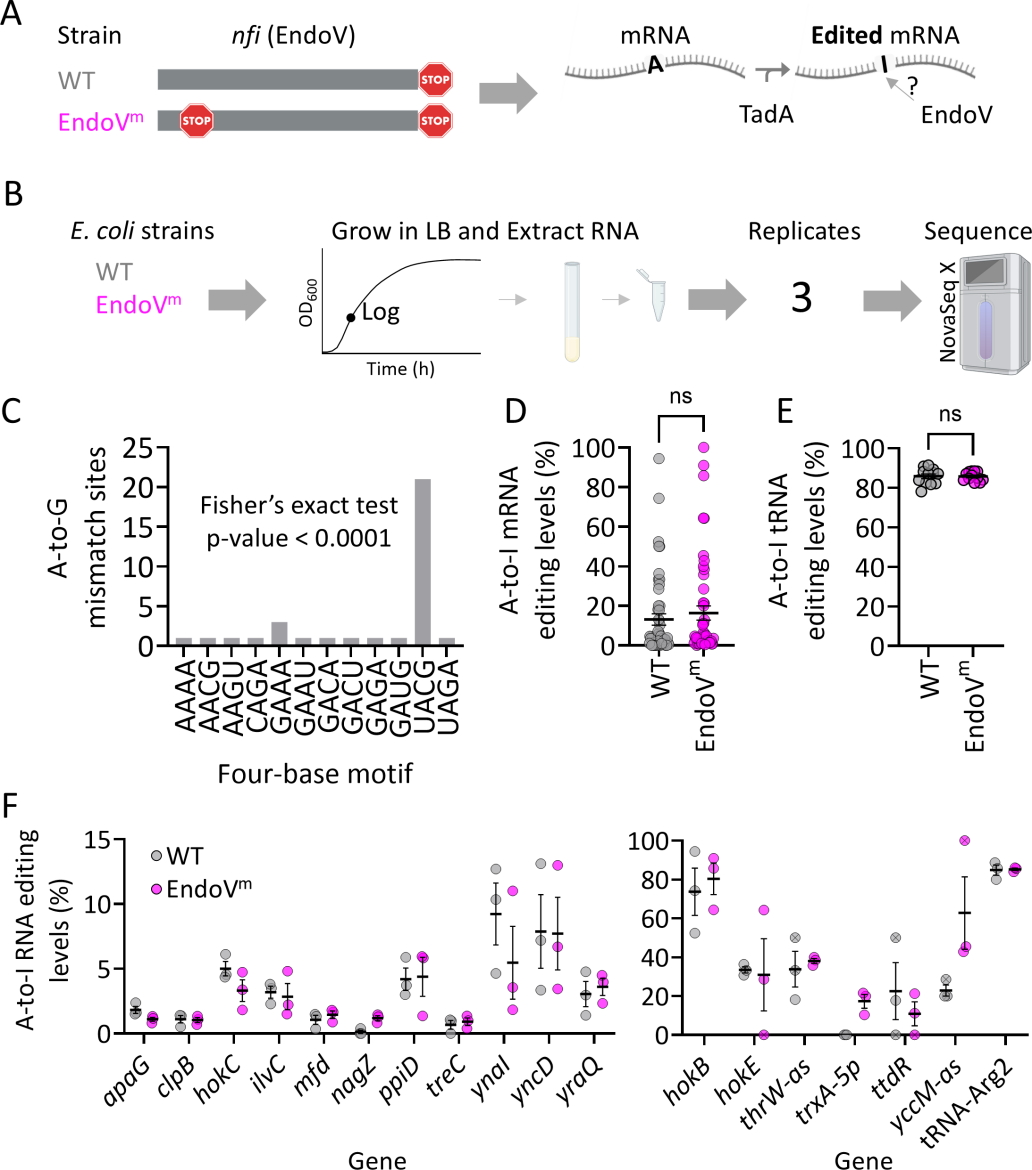
Introducing a premature stop codon at EndoV does not affect A-to-I RNA editing occurrence or levels. (**A**) Research question and a schematic description of the chromosomal STOP mutation in the *nfi* gene. (**B**) Experimental design: WT and EndoV^m^ strains of *E. coli* were grown at 34°C in LB to mid-logarithmic phase. RNA was collected in three biological replicates (the entire experiment was repeated three times on different days). (**C**) RNA-DNA A-to-G mismatches (candidate A-to-I editing sites) are significantly enriched in the UACG motif (Fisher’s exact test; *P* < 0.0001). (**D**) A-to-I mRNA editing levels in the WT and EndoV^m^ strains in LB. (**E**) A-to-I tRNA editing levels in the WT and EndoV^m^ strains in LB. (**F**) An overview of RNA editing levels in identified transcripts. On the left panel are sites with an average editing of below 20%. On the right panel are sites with an average editing of above 20%. Data points marked with “x” have read coverage lower than 10 reads. All data are found in Tables S2–S5. Statistical analysis in panels D and E was conducted using an unpaired Student’s *t*-test. In panel F, we conducted an unpaired Student’s *t*-test between the editing values of each gene separately and then applied a false discovery rate (FDR) correction for multiple testing (Benjamini-Hochberg). No significant difference (*P* ≤ 0.01) was discovered after applying the FDR correction.

First, we aimed to examine whether EndoV loss-of-function affects RNA editing *in vivo* in *E. coli*. For this purpose, we created a mutant strain (EndoV^m^) with a premature stop codon. We grew EndoV^m^ alongside its WT parent strain, extracted RNA, and sent it for RNA-seq (Fig. 1A and B). Importantly, while EndoV protein levels were not measured, the premature stop codon represents a presumed strong loss-of-function mutation. Moreover, we observed that *nfi* expression was significantly reduced in the EndoV^m^ compared to the WT strain (3.39-fold decrease; Table S1), supporting a substantial loss of EndoV function in the mutant. Because inosine is identified as guanosine during cDNA synthesis, we focused on A-to-G mismatches between the RNA-seq and the reference genome. We detected 34 A-to-G mismatched sites, with significant enrichment in the canonical UACG motif of TadA (Fig. 1C; Tables S2 and S3). Importantly, 20/21 sites within the UACG motif have been reported previously, validating our analysis (11–13, 23). No significant difference in the average global mRNA or tRNA editing levels was observed between the two strains (Fig. 1D and E; Table S4). Furthermore, we did not observe a significant difference in editing levels or RNA expression of individual mRNAs between the WT and EndoV^m^ strains, excluding the possibility that endogenous EndoV expression regulates a subset of identified editing events (Fig. 1F; Tables S1 and S5). Finally, Sanger sequencing of the highly edited *hokB* mRNA and tRNA-Arg2 did not reveal a visible difference in editing levels between the WT and EndoV^m^ strains (Fig. S1). Thus, introducing a premature stop codon does not affect *in vivo* editing levels in *E. coli* under exponential-growth conditions in rich medium.

### Overexpressing EndoV does not affect *in vivo* A-to-I RNA editing occurrence or levels

**Fig 2:**
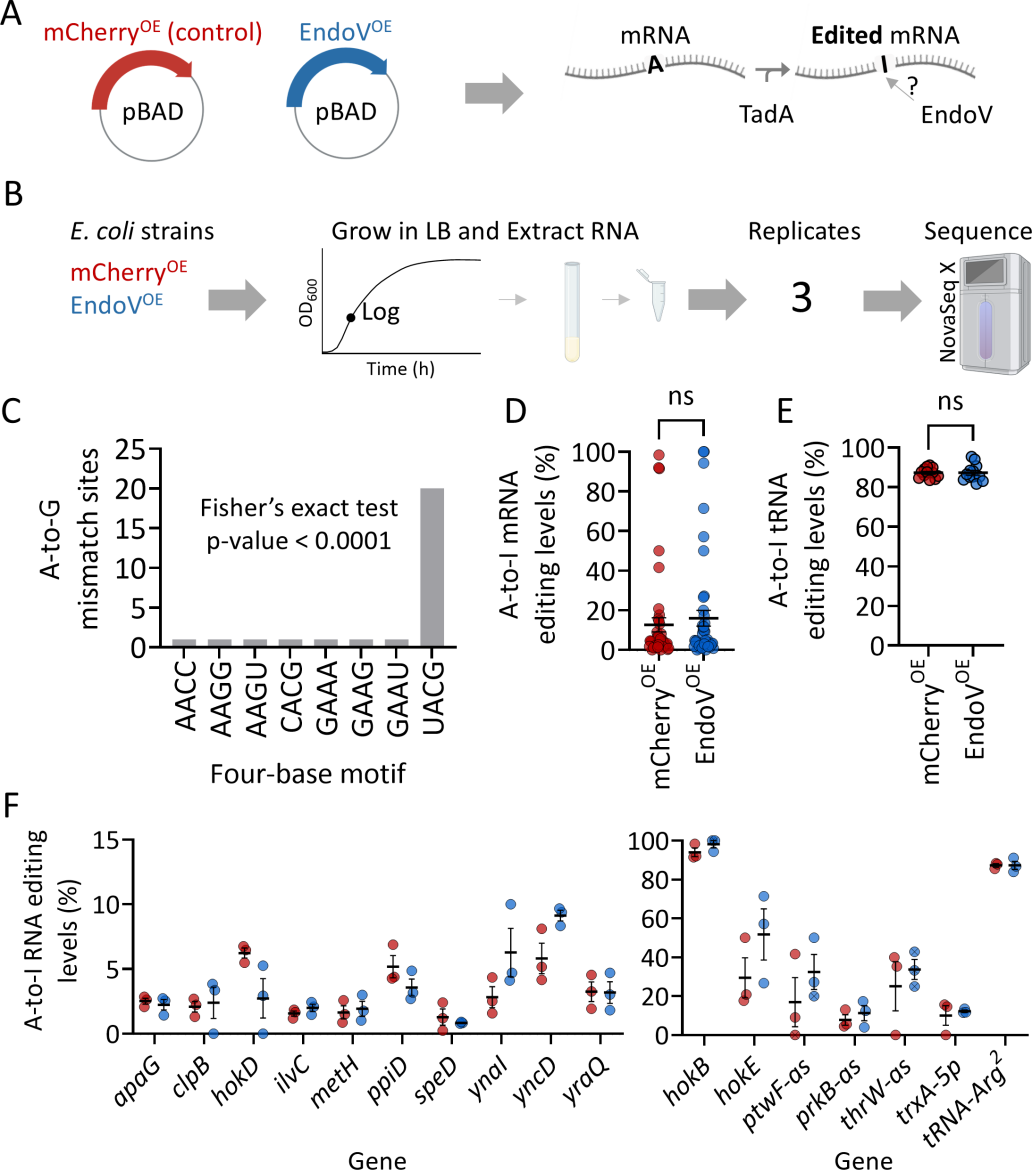
Overexpressing EndoV does not affect *in vivo* A-to-I RNA editing occurrence or levels. (**A**) Research question and a schematic description of the plasmid used for overexpressing mCherry (control) or EndoV. (**B**) Experimental design: mCherry^OE^ and EndoV^OE^ strains of *E. coli* were grown at 37°C in LB to mid-logarithmic phase. RNA was collected in three biological replicates (the entire experiment was repeated three times on different days). (**C**). RNA-DNA A-to-G mismatches (candidate A-to-I editing sites) are significantly enriched in the UACG motif (Fisher’s exact test; *P* < 0.0001). (**D**) A-to-I mRNA editing levels in the mCherry^OE^ and EndoV^OE^ strains in LB. (**E**) A-to-I tRNA editing levels in the mCherry^OE^ and EndoV^OE^ strains in LB. (**F**) An overview of RNA editing levels in identified transcripts. On the left panel are sites with an average editing of below 20%. On the right panel are sites with an average editing of above 20%. Data points marked with “x” have read coverage lower than 10 reads. All data are found in Tables S7–S10. Statistical analysis in panels D and E was conducted using an unpaired Student’s *t*-test. In panel F, we conducted an unpaired Student’s *t*-test between the editing values of each gene separately and then applied a false discovery rate correction for multiple testing (Benjamini-Hochberg). No significant difference (*P* ≤ 0.01) was discovered after applying the FDR correction.

Next, we aimed to determine whether overexpression of EndoV affects RNA editing *in vivo* in *E. coli*. For this purpose, we overexpressed EndoV (EndoV^OE^) or mCherry (mCherry^OE^) as a control, extracted RNA, and sent it for RNA-seq (Fig. 2A and B). First, differential gene expression analysis supported overexpression of the *nfi* transcript (141-fold increase) in the EndoV^OE^ compared to the mCherry^OE^ strain (Fig. S2; Table S6). Focusing on A-to-G mismatches between the RNA-seq data and the reference genome, we detected 27 sites with significant enrichment in the canonical UACG motif of TadA (Fig. 2C; Tables S7 and S8). Importantly, all 20 sites within the UACG motif have been reported previously, validating our analysis (11–13, 23). No significant difference in the average global mRNA or tRNA editing levels was observed between the mCherry^OE^ and EndoV^OE^ strains (Fig. 2D and E; Table S9). Furthermore, we did not observe a significant difference in editing levels or RNA expression of individual mRNAs between the mCherry^OE^ and EndoV^OE^ strains, excluding the possibility that overexpressing EndoV regulates a subset of identified editing events (Fig. 2F; Tables S6 and S10). Finally, Sanger sequencing of the highly edited *hokB* mRNA and tRNA-Arg2 did not reveal a visible difference in editing levels between the WT and EndoV^OE^ strains (Fig. S3). Thus, overexpression of EndoV does not affect *in vivo* editing levels in *E. coli* under exponential-growth conditions in rich medium.

## DISCUSSION

Our results show that EndoV does not measurably affect TadA-dependent A-to-I editing frequencies at known sites in *E. coli* under the conditions tested. Although *E. coli* EndoV was previously shown to cleave inosine-containing RNA *in vitro*, we detected no effect on the occurrence or levels of A-to-I editing events *in vivo*, or the expression of edited transcripts, under the conditions tested. The lack of an effect in both EndoV loss-of-function and overexpression strains argues against a major role for EndoV either in regulating the editing reaction itself or in shaping the steady-state pool of edited transcripts during growth in rich medium.

We cannot exclude the possibility that EndoV acts on inosine-containing RNA under other physiological conditions. Given EndoV’s known association with inosine-containing nucleic acids and nucleic acid repair, it remains possible that EndoV may act under stress or damage-related conditions. However, within the experimental framework used here, EndoV does not appear to shape the A-to-I editing landscape in *E. coli*. This finding is important because it rules out a plausible candidate effector of bacterial RNA editing and narrows the search for other factors that influence the occurrence and fate of edited transcripts.

## MATERIALS AND METHODS

### Bacterial strains and plasmid construction

In the current work, we used the MG1655-EcM2.1 (WT and EndoV^m^) and DH10B (mCherry^OE^ and EndoV^OE^) *E. coli* strains. Importantly, both MG1655 and DH10B are laboratory derivatives within the *E. coli* K-12 lineage, and thus we do not expect a strain-specific EndoV activity that could affect the interpretation of our results. The EndoV^m^ strain, with a premature stop codon replacing a serine with a stop codon at the fifth codon of *nfi*, was created using Multiplex Automated Genome Engineering (MAGE), which carries a loss-of-function mutation in *mutS* (24). To mutate *nfi* in *E. coli*, we performed one successive MAGE cycle. We used five 90-base single-stranded oligonucleotides with two phosphorothioate bonds between the last three bases of the 5′ and 3′ ends (CGCGGATCACAGAAGAAGCCAGTTCGATTTGTTGAGCGCGTAATTACGCGAGATCCATAATCACTCCTTAGCGGTGATACTGTTCAGACA) to target the lagging strand in the *nfi* gene. Briefly, cells were grown overnight at 34°C. Then, 30 µL of the saturated culture was transferred into 3 mL of fresh LB medium in a 12 mL tube until reaching an OD_600_ of 0.5 (measured in a 1 cm cuvette in this section) and then moved to a shaking incubator in an Erlenmeyer containing 200 mL of water (200 RPM) at 42°C for 15 min, after which it was moved immediately to ice. Next, 1 mL was transferred to an Eppendorf tube, and the cells were washed twice with ice-cold double-distilled water (DDW) at 13,000 × *g* for 30 s. Next, the bacterial pellet was dissolved in 50 µL of DDW containing 2 µM of the SS-DNA oligo and transferred to a cuvette. Electroporation was performed at 1.78 kV, 200 ohms, and 25 µF. After electroporation, the bacteria were transferred into 1 mL of fresh LB and incubated at 34°C for 4 h. Then, they were diluted 1:10^−3^ and plated in 25 µL on LB-agar-ampicillin plates (100 µg/mL). To identify positive MAGE colonies (referred to as bacterial strains throughout the text), we PCR-amplified fragments encompassing the *E. coli* genomic region with primers corresponding to the mutated and WT forms (differing in one base in their 3′ ends; primer-WT: GAGTGATTATGGATCTCGCGTC; primer-mutant: GAGTGATTATGGATCTCGCGTA; reverse primer: CGTTTTTTCGCCACGCCAATG). Successful/strong PCR amplification implies successful MAGE mutagenesis. Positive colonies were PCR-amplified and sequenced using Sanger sequencing (primers: ATGTCTTTAACCTTGGTCACG and CGTTTTTTCGCCACGCCAATG).

To overexpress EndoV, we cloned *nfi* into an arabinose-inducible plasmid (pBAD), as previously described (14). To clone *nfi*, we PCR-amplified the plasmid backbone (primers: TAAAAGCTTGGGCCCGAAC and CATGGTTAATTCCTCCTGTTAG) and the *nfi* gene (from the *E. coli* chromosome of NC_000913.3; primers: CTAACAGGAGGAATTAACCATGGATCTCGCGTCATTACG and GTTCGGGCCCAAGCTTTTAGGGCTGATTTGCTGTATAG). PCR amplification was made using high-fidelity Kapa DNA polymerase (ROCHE-07958935001). Following PCR, products were visualized on a 1% agarose gel and cleaned using the DNA Clean & Concentrator Kit (Zymo ZR-D4003). Next, products were fused using the In-Fusion Snap Assembly system (Takara #638947) and treated with DPN1 (NEB-R0176S) to eliminate any original plasmid remains that were used as a template for the PCR reaction of the plasmid backbone. All enzymatic reactions were conducted according to the manufacturer’s instructions and carried out with the appropriate NEB buffers for each enzyme. The new plasmid versions were first transformed into DH5α Pir *E. coli*. Next, the plasmid was extracted using the ZymoPURE Plasmid MiniPrep Kit (#ZR-D4209), sequenced to confirm successful construction, and transformed into the DH10B strain. The DH10B strain overexpressing mCherry was used in previous studies (14, 25).

### RNA extractions and cDNA synthesis

Bacteria were grown in LB medium (10 g tryptone, 10 g sodium chloride, and 5 g yeast extract per liter) supplemented with 100 µg/mL ampicillin. The MG1655-EcM2.1 strains (WT and EndoV^m^) grew at 34°C and the DH10B (mCherry^OE^ and EndoV^OE^) at 37°C. For the mCherry^OE^ and EndoV^OE^ strains, arabinose was added at the beginning of the experiment at a final concentration of 0.2%. At mid-log (OD_600_ of 0.5–0.68), 1 mL was collected for RNA extraction. RNA was extracted using the GeneJET RNA Purification Kit (Thermo Scientific #K0731). RNA samples were treated with 4 units of DNase I (NEB #M0303L) for 20 min at 37°C. Finally, after a 15 min incubation at 65°C, cDNA synthesis was performed using GoScript Reverse Transcription Mix (Promega #A2801). To synthesize cDNA, 500 ng of total RNA from *E. coli* strains was primed with random hexamers and reverse-transcribed using the GoScript Reverse Transcription Mix Kit (A2801, Promega) according to the manufacturer’s protocol.

### RNA-seq and RNA editing analysis

We used RNA-seq to sequence three biological replicates collected on different days for the WT, EndoV^m^, mCherry^OE^, and EndoV^OE^ strains. Ribosomal RNA was depleted using the NEBNext rRNA Depletion Kit (Bacteria) (New England Biolabs, #E7850). RNA-seq libraries were constructed using the NEBNext Ultra II Directional RNA Library Prep Kit for Illumina (New England Biolabs, #E7760). DNA-seq libraries were prepared using the NEBNext Ultra II FS DNA Library Prep Kit for Illumina (New England Biolabs, #E7805L). Finally, RNA-seq libraries were sequenced on the NovaSeq X platform (Illumina).

We used CLC Genomics Workbench for all analysis steps (described below).

RNA-seq reads were first trimmed according to length and quality scores to ensure high quality of the reads by using the following parameters: Trim using quality scores = Yes; Quality limit = 0.01; Trim ambiguous nucleotides = Yes; Maximum number of ambiguities = 1; Automatic read-through adapter trimming = Yes; Minimum length = 50; Maximum length = 150; Remove 5′ terminal nucleotides = No; Remove 3′ terminal nucleotides = No; Remove on first read = Yes; Remove on second read (for paired reads) = Yes; Trim to a fixed length = No; Trim end = Trim from 3′-end; Discard short reads = Yes; Discard long reads = No; Save discarded sequences = No; Save broken pairs = No.

Paired RNA reads were merged into a single longer read, maintaining the original orientation of the R1 reads (reverse to the original transcript direction). The parameters were as follows: Mismatch cost = 2; Minimum score = 8; Gap cost = 3; Maximum unaligned end mismatches = 0.

Next, RNA-seq reads were mapped to the NC_000913.3 *E. coli* reference genome with the following parameters: Masking mode = No masking; Match score = 1; Mismatch cost = 2; Cost of insertions and deletions = Linear gap cost; Insertion cost = 3; Deletion cost = 3; Length fraction = 0.95; Similarity fraction = 0.95; Global alignment = No; Non-specific match handling = Ignore.

Initial variant calling was performed using the following parameters: Ignore positions with coverage above = 100,000; Restrict calling to target regions = Not set; Ignore broken pairs = Yes; Ignore non-specific matches = Reads; Minimum coverage = 4; Minimum count = 2; Minimum variant frequency (%) =0.1; Base quality filter = Yes; Neighborhood radius = 5; Minimum central quality = 30; Minimum neighborhood quality = 30; Read direction filter = No; Relative read direction filter = No; Read position filter = No.

After initial variant calling, additional filtering was applied. All filtered variants were required to match all the following criteria: Criteria = “Type contains SNV”; Criteria = “Reference allele contains No”; Criteria = “Frequency (editing level) >= 1%”; Criteria = “# unique start positions >= 3”; Criteria = “# unique end positions >= 3.”

For sites encoded from the positive strand: Criteria = “Reverse read count >= 3”; Criteria = “Reverse read coverage >= 10”; Criteria = “Reference contains A, and the variant contains G.”

For sites encoded from the negative strand: Criteria = “Forward read count >= 3”; Criteria = “Forward read coverage >= 10”; Criteria = “Reference contains T, and the variant contains C.”

Next, we filtered variants shared between at least two biological replicates or samples.

Finally, we extracted the status of the identified shared variants from the initial variants list or the RNA mapping step to ensure we did not miss variants due to our filters and that, when a variant is absent in our initial analysis, it is not because its transcript was not sequenced.

### Sanger sequencing

To examine the RNA editing state by Sanger sequencing in *hokB* and (tRNA-Arg2) *argV*, we performed PCR as described above with specific primers for *hokB* (CGAAAAATTCTGGAAAACAGCACGC and GTTTATGGGAACGCTTGTTTCC) and *argV* (AGTATGCGGTCAAAAGCTGC and GAAGGTTACGGAGAATATGG).

### Differential gene expression analysis

First, to obtain normalized expression measurements (as transcripts per million [TPM]), we mapped the RNA-seq reads (following quality control and trimming of adapter read-through) to the transcriptome of the *E. coli* genome (NC_000913.3) as done previously (13). This transcriptome version contains transcripts that represent *trxA-5p*, *ycjX-5p*, *thrW-as*, and *ptwF-as* because these were not annotated in the original genome. We used the following parameters in CLC Genomics Workbench: Use spike-in controls = No; Mismatch cost = 2; Insertion cost = 3; Deletion cost = 3; Length fraction = 0.5; Similarity fraction = 0.8; Global alignment = No; Strand specific = Reverse; Library type = Bulk; Maximum number of hits for a read = 10; Count paired reads as two = No; Ignore broken pairs = Yes; Expression value = TPM. Finally, we analyzed differential gene expression with default parameters (also using CLC Genomics Workbench).

Genes were considered differentially expressed if they had an FDR-adjusted *P* value ≤0.01. The only exception was nfi in the EndoV^m^ strain (Table S1): we initially tested nfi expression alone, before performing a genome-wide differential expression analysis that required FDR correction.

## Supplementary Material

Reviewer comments

## Data Availability

The RNA-seq data were deposited in the NCBI SRA under accession PRJNA1453721.

## SUPPLEMENTAL MATERIAL

Supplemental figures and legends spectrum.01302-26-s0001.docx Fig. S1 to S3 and Legends for Tables S1 to S10.

Supplemental tables spectrum.01302-26-s0002.xlsx Tables S1 to S10.

OPEN PEER REVIEW reviewer-comments.pdf An accounting of the reviewer comments and feedback.

ASM does not own the copyrights to Supplemental Material that may be linked to, or accessed through, an article. The authors have granted ASM a non-exclusive, world-wide license to publish the Supplemental Material files. Please contact the corresponding author directly for reuse.
