# Supplementary material for: EndoV does not measurably affect TadA-dependent A-to-I RNA editing in Escherichia coli under exponential-growth conditions in rich medium
Source: Microbiol Spectr. 2026 Jul 6;14(8):e01302-26. doi: 10.1128/spectrum.01302-26 (PMC13436118; doi:10.1128/spectrum.01302-26)
Supplement: Supplementary file 1 [file reviewer-comments.pdf]

AMERICAN SOCIETY FOR MICROBIOLOGY | Microbiology Spectrum

EndoV does not measurably affect TadA-dependent A-to-I RNA editing in *Escherichia coli* under exponential-growth conditions in rich medium

Ofir Fargeon and Dan Bar Yaacov

Corresponding Author(s): Dan Bar Yaacov, Ben-Gurion University of the Negev

Review Timeline:

Submission Date:	April 22, 2026
Editorial Decision:	May 25, 2026
Revision Received:	June 9, 2026
Accepted:	June 11, 2026

Editor: Tino Polen

Reviewer(s): Disclosure of reviewer identity is with reference to reviewer comments included in decision letter(s). The following individuals involved in review of your submission have agreed to reveal their identity: Huiquan Liu (Reviewer #1)

Transaction Report:

(Note: With the exception of the correction of typographical or spelling errors that could be a source of ambiguity, letters and reports are not edited. The original formatting of letters and referee reports may not be reflected in this compilation.)

DOI: https://doi.org/10.1128/spectrum.01302-26

Re: Spectrum01302-26 (**Endonuclease V does not regulate *in vivo* A-to-I RNA editing in *Escherichia coli***)

Dear Dr. Dan Bar-Yaacov,

thank you for submitting your manuscript to Microbiology Spectrum.

I received expert reviewer comments on your manuscript which are generally positive, yet also point out the need for improvements and suggest some modifications.
I do hope you find these comments below helpful to improve your manuscript and look forward to receiving a revised version from you.

Please return the manuscript within 60 days; if you cannot complete the modification within this time period, please contact me. If you do not wish to modify the manuscript and prefer to submit it to another journal, notify me immediately so that the manuscript may be formally withdrawn from consideration by Spectrum.

Revision Guidelines
To submit your modified manuscript, log into the submission site at https://spectrum.msubmit.net/cgi-bin/main.plex. Go to Author Tasks and click the appropriate manuscript title to begin. The information you entered when you first submitted the paper will be displayed; update this as necessary. Note the following requirements:

• Upload point-by-point responses to the issues raised by the reviewers in a file named "Response to Reviewers," NOT in your cover letter.
• Upload a compare copy of the manuscript (without figures) as a "Marked-Up Manuscript" file.
• Upload a clean .DOC/.DOCX version of the revised manuscript and remove the previous version.
• Each figure must be uploaded as a separate, editable, high-resolution file (TIFF or EPS preferred), and any multipanel figures must be assembled into one file.
• Any supplemental material intended for posting by ASM should be uploaded with their legends separate from the main manuscript. You can combine all supplemental material into one file (preferred) or split it into a maximum of 10 files with all associated legends included.

For complete guidelines on revision requirements, see our Submission and Review Process webpage. Submission of a paper that does not conform to guidelines may delay acceptance of your manuscript.

Data availability: ASM policy requires that data be available to the public upon online posting of the article, so please verify all links to sequence records, if present, and make sure that each number retrieves the full record of the data. If a new accession number is not linked or a link is broken, provide Spectrum production staff with the correct URL for the record. If the accession numbers for new data are not publicly accessible before the expected online posting of the article, publication may be delayed; please contact production staff (Spectrum@asmusa.org) immediately with the expected release date.

Publication Fees: For information on publication fees and which article types are subject to charges, visit our website. If your manuscript is accepted for publication and any fees apply, you will be contacted separately about payment during the production process; please follow the instructions in that e-mail. Arrangements for payment must be made before your article is published.

ASM Membership: Corresponding authors may join or renew ASM membership to obtain discounts on publication fees. Need to upgrade your membership level? Please contact Customer Service at Service@asmusa.org.

The ASM Journals program strives for constant improvement in our submission and publication process. Please tell us how we can improve your experience by taking this quick Author Survey.

Thank you for submitting your paper to Spectrum.

Sincerely,
Tino Polen
Editor
Microbiology Spectrum

Reviewer #1 (Comments for the Author):

This manuscript addresses an interesting and relevant question: whether Endonuclease V, encoded by nfi, regulates TadA-dependent A-to-I RNA editing in Escherichia coli. The authors combine an nfi loss-of-function mutant, nfi overexpression, and transcriptome-wide RNA-seq analysis. The experimental logic is clear, and the negative result is potentially useful, as it helps

exclude EndoV as a major regulator of detectable A-to-I RNA editing under the tested conditions.

The study is well motivated and the negative result is useful, but the authors should moderate the title, abstract, and conclusions, and more clearly acknowledge the limitations of the mutant design, overexpression validation, and restricted growth conditions. The data support the conclusion that EndoV does not measurably affect TadA-dependent A-to-I RNA editing under the tested conditions, rather than definitively excluding EndoV as a broader regulator of bacterial RNA editing.

Major Comments

1. The title, abstract, and discussion imply that EndoV does not regulate A-to-I RNA editing in E. coli in general. However, the data support a narrower conclusion: EndoV does not measurably affect the detected TadA-dependent editing events under the specific laboratory conditions tested. The authors should revise the wording throughout the manuscript to avoid overgeneralization. For example, a more accurate conclusion would be:"EndoV does not measurably affect TadA-dependent A-to-I RNA editing in E. coli under the tested exponential-growth conditions."

2. The authors introduced a premature stop codon near the beginning of nfi. This is likely to strongly impair EndoV function, but the manuscript does not directly show loss of EndoV protein or enzymatic activity. If additional experiments are not feasible, the manuscript should explicitly state this limitation and describe the allele as a presumed strong loss-of-function mutant rather than definitive proof of complete EndoV absence.

3. The overexpression experiment shows a strong increase in nfi mRNA, but it does not confirm increased EndoV protein level or activity. Since the interpretation depends on increased EndoV function, the authors should either provide protein/activity validation or revise the wording to make clear that overexpression was confirmed at the transcript level only.

4. All experiments were performed under standard rich-medium exponential-growth conditions. This limits the ability to exclude context-dependent roles of EndoV. Given EndoV's known association with inosine-containing nucleic acids and nucleic acid repair, it remains possible that EndoV may act under stress or damage-related conditions. The authors should either test additional conditions or clearly acknowledge this limitation.

5. EndoV would likely act downstream of RNA editing by recognizing or cleaving inosine-containing RNA, rather than directly controlling TadA-mediated deamination. Therefore, unchanged editing percentages may not fully exclude effects on edited RNA stability or processing. If possible, transcript abundance or local read-coverage around edited sites could be examined. Otherwise, this limitation should be mentioned.

Minor Comments

The authors should clarify whether the different strain backgrounds used for the loss-of-function and overexpression experiments could affect interpretation.

The statistical analysis of differential editing should be described more clearly, including coverage thresholds, replicate variability, and how low-coverage sites were handled.

Sanger sequencing results should be presented as supportive validation, since chromatograms are not highly quantitative for measuring modest changes in editing levels.

The manuscript should consistently distinguish between regulation of the editing reaction itself and regulation of the fate of edited RNAs.

Re: Spectrum01302-26 response to reviewers comments

Dear Prof. Polen, Editor, Microbiology Spectrum.

We sincerely thank you for the opportunity to publish our work in Microbiology Spectrum. We are grateful to you and the reviewer for your positive feedback, thoughtful comments, and recognition of the significance of our study.

Please find below our detailed, point-by-point responses to the reviewer comments (in blue).

We look forward to hearing from you.

Best regards,

Dan Bar Yaacov

Reviewer #1 (Comments for the Author):

This manuscript addresses an interesting and relevant question: whether Endonuclease V, encoded by nfi, regulates TadA-dependent A-to-I RNA editing in Escherichia coli. The authors combine an nfi loss-of-function mutant, nfi overexpression, and transcriptome-wide RNA-seq analysis. The experimental logic is clear, and the negative result is potentially useful, as it helps exclude EndoV as a major regulator of detectable A-to-I RNA editing under the tested conditions.

The study is well motivated and the negative result is useful, but the authors should moderate the title, abstract, and conclusions, and more clearly acknowledge the limitations of the mutant design, overexpression validation, and restricted growth conditions. The data support the conclusion that EndoV does not measurably affect TadA-dependent A-to-I RNA editing under the tested conditions, rather than definitively excluding EndoV as a broader regulator of bacterial RNA editing.

Major Comments

1. The title, abstract, and discussion imply that EndoV does not regulate A-to-I RNA editing in E. coli in general. However, the data support a narrower conclusion: EndoV does not measurably affect the detected TadA-dependent editing events under the specific laboratory conditions tested. The authors should revise the wording throughout the manuscript to avoid overgeneralization. For example, a more accurate conclusion would be:"EndoV does not measurably affect TadA-dependent A-to-I RNA editing in E. coli under the tested exponential-growth conditions."

> We agree. We now changed the title to: "EndoV does not measurably affect TadA-dependent A-to-I RNA editing in *Escherichia coli* under exponential-growth conditions in rich medium".

We also revised the abstract

We also went over the text and made sure that the above conclusion is explicitly written.

In the results in the section dealing with the WT and EndoV^{m} strains we change (underlined): "Thus, introducing a premature stop codon does not affect *in vivo* editing levels in *E. coli* under standard laboratory conditions" to "Thus, introducing a premature stop codon does not affect *in vivo* editing levels in *E. coli* under exponential-growth conditions in rich medium."

In the results in the section dealing with the mCherry^{OE} and EndoV^{OE} strains we change (underlined): "Thus, overexpression of EndoV does not affect *in vivo* editing levels in *E. coli* under standard laboratory conditions." to "Thus, overexpression of EndoV does not affect *in vivo* editing levels in *E. coli* under exponential-growth conditions in rich medium."

Notably, we also better emphasized in the discussion that our results are specific to the tested conditions and that other physiological conditions might allow EndoV to process inosine-containing mRNAs (new text is underlined).

"We cannot exclude the possibility that EndoV acts on inosine-containing RNA under other physiological conditions. Given EndoV's known association with inosine-containing nucleic acids and nucleic acid repair, it remains possible that EndoV may act under stress or damage-related conditions. However, within the experimental framework used here, EndoV does not appear to shape the A-to-I editing landscape in *E. coli*."

2. The authors introduced a premature stop codon near the beginning of nfi. This is likely to strongly impair EndoV function, but the manuscript does not directly show loss of EndoV protein or enzymatic activity. If additional experiments are not feasible, the manuscript should explicitly state this limitation and describe the allele as a presumed strong loss-of-function mutant rather than definitive proof of complete EndoV absence.

> Thank you for this comment. We now clearly state that the allele is a presumed strong loss-of-function mutant. Moreover, our RNA-seq analysis also revealed that *nfi* expression in the EndoV^{m} strain was significantly reduced, further supporting the loss of function phenotype.

We added the following text:

"Importantly, while EndoV protein levels were not measured, the premature stop codon represents a presumed strong loss-of-function mutation. Moreover, we observed that *nfi* expression was significantly reduced in the EndoV^{m} compared to the WT strain (3.39-fold decrease; Table S1), supporting a substantial loss of EndoV function in the mutant."

3. The overexpression experiment shows a strong increase in nfi mRNA, but it does not confirm increased EndoV protein level or activity. Since the interpretation depends on increased EndoV function, the authors should either provide protein/activity validation or revise the wording to make clear that overexpression was confirmed at the transcript level only.

> Thank you for this comment. We now revised the wording to make clear that overexpression was confirmed at the transcript level only.

We changed the following text (deleted – strikethrough; new – underlined):

"First, differential gene expression analysis supported overexpression of ~~EndoV~~ the *nfi* transcript (146-fold increase) in the EndoVOE compared to the mCherryOE strain (Figure S2 and Table S5).."

4. All experiments were performed under standard rich-medium exponential-growth conditions. This limits the ability to exclude context-dependent roles of EndoV. Given EndoV's known association with inosine-containing nucleic acids and nucleic acid repair, it remains possible that EndoV may act under stress or damage-related conditions. The authors should either test additional conditions or clearly acknowledge this limitation.

> Thank you for this comment. We agree and indeed acknowledge this limitation in the discussion (see below). In addition, we now specifically added the suggested text by the reviewer (underlined):

"We cannot exclude the possibility that EndoV acts on inosine-containing RNA under other physiological conditions. However, within the experimental framework used here, EndoV does not appear to shape the A-to-I editing landscape in E. coli. Given EndoV's known association with inosine-containing nucleic acids and nucleic acid repair, it remains possible that EndoV may act under stress or damage-related conditions"

5. EndoV would likely act downstream of RNA editing by recognizing or cleaving inosine-containing RNA, rather than directly controlling TadA-mediated deamination. Therefore, unchanged editing percentages may not fully exclude effects on edited RNA stability or processing. If possible, transcript abundance or local read-coverage around edited sites could be examined. Otherwise, this limitation should be mentioned.

> Thank you for this comment. We used our RNA-seq data to conduct differential gene expression analysis. We found that edited transcripts (including tRNA-Arg2) did not show significant differences in expression in either EndoV^{m} or EndoV^{OE} strains compared to their controls. Thus, it seems there is no effect on the steady-state level of edited transcripts in the strains we created.

To communicate this analysis and result, we added a new Table S5, revised Tables S6 (formerly Table S5) and Supplementary Figure 2, and added the relevant text.

Below are the changes we made to the text. New text is underlined, and deleted text is strikethrough.

In the results:

"Furthermore, we did not observe a significant difference in editing levels or RNA expression of individual mRNAs between the WT and EndoVm strains, excluding the possibility that endogenous EndoV expression regulates a subset of identified editing events (Figure 1F, ~~and~~ Table S4, and Table S5)."

"Furthermore, we did not observe a significant difference in editing levels or RNA expression of individual mRNAs between the mCherry^{OE} and EndoV^{OE} strains, excluding the possibility that overexpressing EndoV regulates a subset of identified editing events (Figure 2F, Table S6, and ~~Table S9~~Table S10)."

In the discussion:

"Our results show that EndoV does not measurably affect TadA-dependent A-to-I editing frequencies at known sites in *E. coli* under the conditions tested. Although *E. coli* EndoV was previously shown to

cleave inosine-containing RNA *in vitro*, we detected no effect on the occurrence or levels of A-to-I editing events *in vivo*, or the expression of edited transcripts, under the conditions tested. The lack of an effect in both EndoV loss-of-function and overexpression strains argues against a major role for EndoV either in regulating the editing reaction itself or in shaping the steady-state pool of edited transcripts during growth in rich medium."

In the material and methods section:

“Differential gene expression analysis

First, to obtain normalized expression measurements (as transcripts per million – TPM), we mapped the RNA-seq reads (following quality control and trimming of adapter read-through) to the transcriptome of the *E. coli* genome (NC_000913.3) as done previously (13). This transcriptome version contains transcripts that represent *trxA-5p, ycjX-5p, thrW-as*, and *ptwF-as* because these were not annotated in the original genome. We used ~~with its annotated genes with~~ the following parameters in CLC Genomics Workbench: Use spike-in controls = no; Mismatch cost = 2; Insertion cost = 3; Deletion cost = 3; Length fraction = 0.~~8~~5; Similarity fraction = 0.8; Global alignment = No; Strand specific = Reverse; Library type = Bulk; Maximum number of hits for a read = 10; Count paired reads as two = No; Ignore broken pairs = Yes; Expression value = TPM. Finally, we analyzed differential gene expression with default parameters (also using CLC Genomics Workbench).

Genes were considered differentially expressed if they had a false discovery rate (FDR)–adjusted p-value less than or equal to 0.01. The only exception was nfi in the $EndoV^{m}$ strain (Table S1): we initially tested nfi expression alone, before performing a genome-wide differential expression analysis that required FDR correction.”

Minor Comments

The authors should clarify whether the different strain backgrounds used for the loss-of-function and overexpression experiments could affect interpretation.

> Thank you for this comment. Both MG1655 and DH10B are laboratory derivatives within the E. coli K-12 lineage. Also, most of the edited transcripts are shared between the strains. Therefore, the different strain backgrounds used for the loss-of-function and overexpression likely did not affect the interpretation of our results.

In the material and methods section, we added the following text:

"Importantly, both MG1655 and DH10B are laboratory derivatives within the *E. coli* K-12 lineage, and thus we do not expect a strain-specific EndoV activity that could affect the interpretation of our results."

The statistical analysis of differential editing should be described more clearly, including coverage thresholds, replicate variability, and how low-coverage sites were handled.

> Thank you for this comment. We have now marked in figures 1 and 2 sites with coverage lower than 10. The workflow and rational is explained in the material and methods section describing how we detect RNA editing events.

We revised the legends of these figures and also better explained how the statistical analysis of differential editing was conducted.

Figure 1 legend (new text is underlined):
F. An overview of RNA editing levels in identified transcripts. On the left panel are sites with an average editing of below 20%. On the right panel are sites with an average editing of above 20%. Data points marked with "x" have read coverage lower than 10 reads. All data are found in Tables S1-S4. Statistical analysis in panels D and E was conducted using an unpaired Student's t-test. In panel F, we conducted unpaired Student's t-test between the editing values of each gene separately and then applied a false discovery rate correction for multiple testing (Benjamini-Hochberg). No significant difference ($p \leq 0.01$) was discovered after applying the FDR correction.

Figure 2 legend (new text is underlined):

F. An overview of RNA editing levels in identified transcripts. On the left panel are sites with an average editing of below 20%. On the right panel are sites with an average editing of above 20%. Data points marked with "x" have read coverage lower than 10 reads. All data are found in Tables S7-S10. Statistical analysis in panels D and E was conducted using an unpaired Student's t-test. In panel F, we conducted unpaired Student's t-test between the editing values of each gene separately and then applied a false discovery rate correction for multiple testing (Benjamini-Hochberg). No significant difference ($p \leq 0.01$) was discovered after applying the FDR correction.

Sanger sequencing results should be presented as supportive validation, since chromatograms are not highly quantitative for measuring modest changes in editing levels.

> We agree. We added the following text to the legend of Fig S1 and Fig S3: "Notably, Sanger sequencing is presented here only as supportive validation to examine noticeable changes in editing, since chromatograms are not highly quantitative for measuring modest changes in editing levels."

The manuscript should consistently distinguish between regulation of the editing reaction itself and regulation of the fate of edited RNAs.

> Thank you for this comment. We have revised the text as suggested.

In the abstract:

"Here, we examined whether endonuclease V (EndoV; encoded by nfi) cleaves inosine-containing RNAs in vivo and thereby influences the abundance and fate of A-to-I–edited mRNAs in Escherichia coli."

"Our findings suggest that, in contrast to human EndoV, bacterial EndoV does not regulate the steady-state pool of edited mRNAs in *E. coli* under nutrient-rich, exponential-growth conditions *in vivo*."

In the introduction:

"Thus, here, we examined whether EndoV can cleave inosine-containing RNA in vivo and thereby influence the landscape and fate of A-to-I–edited transcripts in bacteria."

In the discussion:

"Our results show that EndoV does not measurably affect TadA-dependent A-to-I editing frequencies at known sites in *E. coli* under the conditions tested. Although *E. coli* EndoV was previously shown to cleave inosine-containing RNA *in vitro*, we detected no effect on the occurrence or levels of A-to-I editing events *in vivo*, or the expression of edited transcripts, under the conditions tested. The lack of an effect in both EndoV loss-of-function and overexpression strains argues against a major role for EndoV either in regulating the editing reaction itself or in shaping the steady-state pool of edited transcripts during growth in rich medium."

Re: Spectrum01302-26R1 (**EndoV does not measurably affect TadA-dependent A-to-I RNA editing in*Escherichia coli* under exponential-growth conditions in rich medium**)

Dear Dr. Dan Bar Yaacov,

your revised manuscript has been accepted, and I am forwarding it to the ASM production staff for publication.
Your paper will first be checked to make sure all elements meet the technical requirements. ASM staff will contact you if anything needs to be revised before copyediting and production can begin. Otherwise, you will be notified when your proofs are ready to be viewed.

Data Availability: ASM policy requires that data be available to the public upon online posting of the article, so please verify all links to sequence records, if present, and make sure that each number retrieves the full record of the data. If a new accession number is not linked or a link is broken, provide production staff with the correct URL for the record. If the accession numbers for new data are not publicly accessible before the expected online posting of the article, publication may be delayed; please contact ASM production staff immediately with the expected release date.

Publication Fees: For information on publication fees and which article types have charges, please visit ourwebsite. We have partnered with Copyright Clearance Center (CCC) to collect author charges. If fees apply to your paper, you will receive a message from no-reply@copyright.com with further instructions. For questions related to paying charges through RightsLink, please contact CCC at ASM_Support@copyright.com or toll free at +1-877-622-5543. CCC makes every attempt to respond to all emails within 24 hours.

ASM Membership: Corresponding authors may join or renew ASM membership to obtain discounts on publication fees. Need to upgrade your membership level? Please contact Customer Service at Service@asmusa.org.

Embargo Policy: A press release may be issued as soon as the manuscript is posted on theSpectrum Latest Articles webpage. The corresponding author will receive an email with the subject line "ASM Journals Author Services Notification" when the article is available online.

The ASM Journals program strives for constant improvement in our submission and publication process. Please tell us how we can improve your experience by taking this quick Author Survey.

Thank you for submitting your paper to Spectrum.

Sincerely,
Tino Polen
Editor
Microbiology Spectrum

Reviewer #1 (Comments for the Author):

The authors have satisfactorily addressed all my previous concerns. I have no further comments.
