# Supplementary material for: EndoV does not measurably affect TadA-dependent A-to-I RNA editing in Escherichia coli under exponential-growth conditions in rich medium
Source: Microbiol Spectr. 2026 Jul 6;14(8):e01302-26. doi: 10.1128/spectrum.01302-26 (PMC13436118; doi:10.1128/spectrum.01302-26)
Supplement: Supplementary file 2 [file spectrum.01302-26-s0001.docx]

**Supplementary Figures**

**Figure S1.** Sanger sequencing of the endogenous *hokB* mRNA and tRNA-Arg2 (encoded by *argV*) in the WT and EndoV^m^ strains. Black arrows mark the double peak of A and G (I) observed in the samples. Sequences were aligned to the *E. coli* reference genome (NC_000913.3). Notably, Sanger sequencing is presented here only as supportive validation to examine noticeable changes in editing, since chromatograms are not highly quantitative for measuring modest changes in editing levels.

**Figure S2. *nfi* is significantly upregulated in the EndoV^OE^ strain.** Volcano plots showing genes with significant change in expression (FDR p-value < 0.01) in the EndoV^OE^ compared to the mCherry^OE^ strains. Notice that *nfi* is the most upregulated gene (marked in red and labeled).

**Figure S3.** Sanger sequencing of the endogenous *hokB* mRNA and tRNA-Arg2 (encoded by *argV*) in the mCherry^OE^ (control) and EndoV^OE^ strains. Black arrows mark the edited site harboring a black peak of G (I) in the samples. Sequences were aligned to the *E. coli* reference genome (NC_000913.3). Notably, Sanger sequencing is presented here only as supportive validation to examine noticeable changes in editing, since chromatograms are not highly quantitative for measuring modest changes in editing levels.

**Tables legends**

**Table S1. Differential gene expression analysis of the WT and EndoV^m^ strains of *E. coli*. Genes with significant differences in expression in the EndoV^m^ compared to the WT strain are marked in red. Transcripts with identified editing are marked in light blue, while *nfi* is marked in yellow.**

**Table S2.** A-to-G RNA–genome mismatches that passed all filtering criteria and were detected in at least two biological replicates or samples in the WT and EndoV^m^ strains.

**Table S3.** Fisher's exact test assessing enrichment of the UACG motif among sites listed in Table S1.

**Table S4.** Editing frequencies of A-to-I sites within the UACG motif in WT and EndoV^m^ strains, including sites that did not pass the final filters but were recovered from the initial variant calling or RNA mapping.

**Table S5.** Statistical analyses of edited sites listed in Table S3 were performed per mRNA using the F-test, Student's t-test, and false discovery rate (FDR) correction for multiple testing.

**Table S6.** **Differential gene expression analysis of the mCherry^OE^ and EndoV^OE^ strains of *E. coli*. Genes with significant differences in expression in the EndoV^m^ compared to the WT strain are marked in red. Transcripts with identified editing are marked in light blue, while *nfi* is marked in yellow.**

**Table S7.** A-to-G RNA–genome mismatches that passed all filtering criteria and were detected in at least two biological replicates or samples in the mCherry^OE^ and EndoV^OE^ strains.

**Table S8.** Fisher's exact test assessing enrichment of the UACG motif among sites listed in Table S6.

**Table S9.** Editing frequencies of A-to-I sites within the UACG motif in mCherry^OE^ and EndoV^OE^ strains, including sites that did not pass the final filters but were recovered from the initial variant calling or RNA mapping.

**Table S10.** Statistical analyses of edited sites listed in Table S9 were performed per mRNA using the F-test, Student's t-test, and FDR correction for multiple testing.
